# Prognostic value of the De Ritis ratio for 1-year major adverse cardiovascular events in elderly patients with acute decompensated heart failure

**DOI:** 10.2478/abm-2026-0012

**Published:** 2026-04-30

**Authors:** Truong Huy Hoang, Dan Van Buu Do, Nguyen Khoi Dang, Thao Le Phuong Nguyen

**Affiliations:** 1 Department of Internal Medicine, Faculty of Medicine, Pham Ngoc Thach University of Medicine, Ho Chi Minh City 700000, Vietnam; 2 Department of Cardiology 5, Tam Duc Cardiology Hospital, Ho Chi Minh City 700000, Vietnam; 3 Department of Cardiac Electrophysiology and Arrhythmia, Tam Duc Cardiology Hospital, Ho Chi Minh City 700000, Vietnam; 4 Outpatient Department, Tam Duc Cardiology Hospital, Ho Chi Minh City 700000, Vietnam

**Keywords:** acute decompensated heart failure, alanine aminotransferase, aspartate aminotransferase, De Ritis ratio, elderly, major adverse cardiovascular events, risk stratification

## Abstract

**Background:**

The De Ritis ratio, derived from serum aspartate aminotransferase and alanine aminotransferase levels, has shown prognostic value in cardiovascular diseases. Its role in elderly patients hospitalized with acute decompensated heart failure (ADHF) remains uncertain.

**Objectives:**

To evaluate the prognostic value of the De Ritis ratio for 1-year major adverse cardiovascular events (MACEs) in elderly patients with ADHF.

**Methods:**

A single-center, prospective cohort of 249 patients aged ≥65 years (median age 78, 53.8% female) hospitalized with ADHF was enrolled. Patients were categorized into tertiles by De Ritis ratio: <1.18 (n = 82), 1.18-1.49 (n = 88), and ≥l.49 (n = 79). The primary outcome was 1-year MACEs, including all-cause death, non-fatal myocardial infarction, stroke, and heart failure (HF) rehospitalization. Cox regression was used to identify independent predictors.

**Results:**

At 1 year, 68 patients (27.3%) died and 150 (60.2%) experienced MACEs. The event rate increased progressively across tertiles: 31.7%, 64.8%, and 84.8% (*P* < 0.001). The De Ritis ratio was significantly associated with age, diabetes mellitus, advanced HF symptoms, N-terminal pro-B-type natriuretic peptide, and reduced left ventricular ejection fraction. The optimal cutoff for predicting MACEs was 1.30 (C-statistic: 0.760, 95% confidence interval [CI]: 0.697-0.823, *P* < 0.001), with 78% sensitivity and 72.7% specificity. A De Ritis ratio >1.30 independently predicted outcomes (hazard ratio: 2.362; 95% CI: 1.536-3.634; *P* < 0.001).

**Conclusions:**

An elevated De Ritis ratio is an independent predictor of 1-year MACEs in elderly patients with ADHF. This simple, accessible biomarker may aid clinical risk stratification.

Acute decompensated heart failure (ADHF) remains a major global health challenge, especially in the elderly population [1-3]. It is associated with high rates of mortality, morbidity, and recurrent hospitalizations. Risk stratification in these patients is challenging due to the heterogeneity of comorbidities and pathophysiological mechanisms [4]. Established prognostic factors in both acute and chronic heart failure (HF) include advanced age, low systolic blood pressure, renal dysfunction, hyponatremia, elevated natriuretic peptides, reduced left ventricular ejection fraction (LVEF), higher New York Heart Association (NYHA) functional class, atrial fibrillation, diabetes, anemia, and chronic lung disease [5, 6]. While biomarkers such as N-terminal pro-B-type natriuretic peptide (NT-proBNP) and high-sensitivity troponin have demonstrated valuable prognostic value in HF, their widespread clinical application remains limited in resource-constrained settings due to high costs and technical demands [7].

The ratio of aspartate aminotransferase (AST) to alanine aminotransferase (ALT), known as the De Ritis ratio, has long been utilized as a hepatic biomarker, especially in alcoholic liver disease and cirrhosis [8]. Recent studies, however, have revealed its association with cardiovascular diseases and allcause mortality in various populations, including patients with acute myocardial infarction (MI), cardiogenic shock, and HF [9-11]. Reported thresholds vary across cohorts: in elderly patients hospitalized with ADHF [10], a ratio ≥1.7 predicted 1-year mortality, whereas in HF with preserved ejection fraction cohort, values >1.0 were linked to adverse outcomes [12]. The underlying mechanisms are hypothesized to involve hepatic congestion, systemic hypoperfusion, and multiorgan dysfunction, conditions frequently encountered in patients with ADHF [13]. Despite this plausible pathophysiological link, limited data exist on the prognostic significance of the De Ritis ratio in elderly patients hospitalized for ADHF. The aim of this study was to investigate the association between the De Ritis ratio at hospital admission and 1-year major adverse cardiovascular events (MACEs) in elderly patients hospitalized with ADHF

## Methods

The study protocol was approved by the Ethics Committee of Pham Ngoc Thach University of Medicine (1008/TĐHYKPNT-HĐĐĐ) and conducted in accordance with the principles outlined in the Declaration of Helsinki. Written informed consent was obtained from all participants.

### Study design and population

This was a single-center, prospective, longitudinal observational cohort study conducted at Tam Duc Cardiology Hospital, Ho Chi Minh City, Vietnam, from December 2020 to August 2023. The study enrolled consecutive patients aged ≥65 years who were hospitalized with ADHF. ADHF was diagnosed based on the 2021 European Society of Cardiology guidelines for the diagnosis and treatment of acute and chronic HF, requiring the presence of at least one symptom and one physical sign of HF, along with elevated NT-proBNP levels (≥300 pg/mL) and echocardiographic evidence of cardiac dysfunction [2]. Exclusion criteria included (1) acute coronary syndrome at admission; (2) elevated aminotransferase levels due to other causes unrelated to HF, such as acute or chronic hepatitis B virus, chronic hepatitis C virus, cirrhosis, acute cholecystitis, or acute pancreatitis; (3) severe renal failure requiring peritoneal dialysis or hemodialysis; (4) acute myocarditis; and (5) active severe systemic infection at admission.

### Data collection

Demographic, clinical, laboratory, and echocardiographic data were collected at baseline. All patients underwent standard investigations, including blood tests, chest radiography, electrocardiography, and transthoracic echocardiography. Biochemical tests, including AST and ALT, were performed from the initial blood sample obtained at hospital admission. Analyses were conducted in the hospital’s laboratory using the Beckman Coulter AU680 analyzer (Beckman Coulter, Japan). Blood samples were processed within 2-3 h of collection, ensuring a storage time of no more than 6 h according to ISO 15189:2012 standards to maintain sample integrity and result accuracy. The reference ranges for AST and ALT in our laboratory were <50 U/L. The De Ritis ratio was defined as the ratio of AST to ALT activity, both measured from the same blood sample obtained at admission. Based on the distribution of De Ritis ratio value in the study population, patients were classified into 3 tertiles: <1.18 (first tertile), 1.18-1.49 (second tertile), and ≥1.49 (third tertile). This classification was applied for comparing clinical characteristics and outcome analyses. Anemia was defined as a hemoglobin concentration of less than 13 g/dL for men or less than 12 g/dL for women [14]. Hypertension was defined as previous history of hypertension, or if the patient was on antihypertensive medication [15]. Diabetes mellitus was diagnosed when fasting blood glucose was ≥126 mg/dL, or HbA1c was ≥6.5%, or if the patient was on antidiabetic medications [16]. Renal function was assessed by calculating the estimated glomerular filtration rate according to the Kidney Disease: Improving Global Outcomes (KDIGO) 2012 guidelines [17].

### Follow-up and outcomes

After discharge, patients were scheduled for follow-up visits at 1 week, 1 month, 3 months, 6 months, and 1 year. Follow-up was conducted via outpatient visits or standardized telephone interviews by 2 trained research physicians. Information collected included survival status, date and cause of death (if applicable), rehospitalizations for HF, nonfatal MI, and stroke. Data were cross-referenced with the hospital’s electronic medical records and official death certificates when available. All discrepancies were resolved by direct contact or document verification. Complete 1-year follow-up data were obtained for all enrolled patients. The primary outcome was the incidence of MACEs within 1 year from the time of index hospital admission, defined as a composite of allcause mortality, nonfatal MI, stroke, and rehospitalization for HF.

### Statistical analysis

All statistical analyses were performed using IBM SPSS Statistics version 25.0 (IBM Corp.). The normality of continuous variables was assessed using the Kolmogorov-Smirnov test. Continuous variables were summarized as median (Me) and interquartile range (IQR), while categorical variables were expressed as frequencies and percentages. Comparisons of baseline demographic, clinical, laboratory, and echocardiographic characteristics across tertiles of the De Ritis ratio were conducted using the chi-squared test or Fisher’s exact test for categorical variables. Pairwise comparisons of proportions were performed using *Z*-tests with Bonferroni adjustment to correct for multiple comparisons. Continuous variables were compared using the Kruskal-Wallis test, and when appropriate, pairwise comparisons were conducted using Mann-Whitney *U* tests with Bonferroni-adjusted significance levels. Due to positive skewness, NT-proBNP levels were log-transformed prior to regression analysis. The association between clinical variables and 1-year MACEs was assessed using univariate and multivariate Cox proportional hazards regression models. Variables with clinical relevance or a *P*-value <0.2 in univariate analysis were included in the multivariate model. Results were presented as hazard ratios (HRs) with corresponding 95% confidence intervals (CIs). Kaplan-Meier survival analysis was performed to estimate event-free survival rates, and differences between tertile groups were assessed using the log-rank test. The prognostic performance of the De Ritis ratio in predicting MACEs was evaluated by constructing a receiver operating characteristic (ROC) curve, with calculation of the area under the curve (AUC), optimal cutoff value, sensitivity, specificity, positive predictive value, and negative predictive value. A two-tailed *P*-value <0.05 was considered statistically significant.

## Results

Baseline characteristics across De Ritis tertiles are shown in **Table 1**. Among 249 patients (median age 78 years; 53.8% female), higher De Ritis tertiles captured a more decompensated phenotype. Age increased across tertiles, and diabetes was more prevalent in the second and third tertiles. Markers of clinical severity and congestion demonstrated stepwise gradients: NYHA III-IV (29.3%, 55.7%, 81.0%; *P* < 0.001), NT-proBNP (median 2,892 → 5,367 → 8,606 pg/mL; *P* < 0.001), larger inferior vena cava (IVC) diameters and more IVC ≥ 21 mm (26.8%, 40.9%, 53.2%; *P* = 0.003), and lower LVEF (median 45% → 42% → 32%; *P* < 0.001). Median AST rose across tertiles (35.5 → 45.0 → 55.0 U/L; *P* < 0.001), while ALT showed a modest decrease from the first to the third tertile (37.0 → 33.0 → 32.0 U/L; *P* = 0.012). Other comorbidities (coronary artery disease [CAD], atrial fibrillation, chronic kidney disease [CKD], anemia, chronic lung disease) were broadly similar across groups.

**Table 1.** Baseline characteristics of patients hospitalized with ADHF according to tertiles of the De Ritis ratio

Variables	Total cohort (n = 249)	De Ritis ratio			*P*
		1st tertile (n = 82)	2nd tertile (n = 88)	3rd tertile (n = 79)	
Age, years, Me (IQR)	78 (70; 85)	74 (70; 81.2)	81.5 (73; 89)	78 (70; 84)	0.003
Female, n (%)	134 (53.8)	42 (51.2)	53 (60.2)	39 (49.4)	0.316
Arterial hypertension, n (%)	215 (86.3)	68 (82.9)	79 (89.8)	68 (86.1)	0.429
CAD, n (%)	157 (63.1)	47 (57.3)	57 (64.8)	53 (67.1)	0.402
Previous MI, n (%)	95 (38.2)	28 (34.1)	36 (40.9)	31 (39.2)	0.644
Dyslipidemia, n (%)	233 (93.6)	75 (91.5)	86 (97.7)	72 (91.1)	0.141
Diabetes mellitus, n (%)	93 (37.3)	22 (26.8)	34 (38.6)	37 (46.8)	0.031
Previous CVA, n (%)	57 (22.9)	13 (15.9)	24 (27.3)	20 (25.3)	0.172
Atrial fibrillation, n (%)	121 (48.6)	39 (47.6)	41 (46.6)	41 (51.9)	0.770
CKD, n (%)	168 (67.5)	53 (64.6)	59 (67)	56 (70.9)	0.695
Chronic lung disease, n (%)	27 (10.8)	10 (12.2)	7 (8.0)	10 (12.7)	0.553
Anemia, n (%)	130 (52.2)	40 (48.8)	45 (51.1)	45 (57)	0.565
Chest pain, n (%)	41 (16.5)	12 (14.6)	13 (14.8)	16 (20.3)	0.547
Dyspnea, n (%)	241 (96.8)	80 (97.6)	85 (96.6)	76 (96.2)	0.880
Peripheral edema, n (%)	71 (28.5)	19 (23.2)	23 (26.1)	29 (36.7)	0.136
NYHA class III-IV, n (%)	137 (55)	24 (29.3)	49 (55.7)	64 (81)	<0.001
Systolic blood pressure, mm Hg, Me (IQR)	120 (110; 138)	121.5 (115; 133.7)	121.5 (110.5; 139.7)	120 (110; 137)	0.640
Diastolic blood pressure, mm Hg, Me (IQR)	70 (61.5; 80)	70 (63; 80)	75 (65; 80)	70 (60; 80)	0.436
Heart rate, bpm, Me (IQR)	89 (82; 100.5)	86.5 (79.7; 100)	89 (80.5; 102.7)	92 (82; 105)	0.313
NT-proBNP, Me (IQR), pg/mL, Me (IQR)	5,211 (2,887; 10,202)	2,892 (2,156; 4,668)	5,367 (3,117; 9,349)	8,606 (5,639; 17,891)	<0.001
Hemoglobin, g/đL, Me (IQR)	12.2 (10.5; 13.8)	12.3 (10.9; 13.8)	12 (10.3; 13.5)	12.0 (10.3; 14)	0.595
Creatinine, μmol/L, Me (IQR)	110 (88; 134.5)	107.5 (91; 127)	114 (84.2; 137.7)	113 (88; 141)	0.791
eGFR, mL/min/1.73 m^2^, Me (IQR)	48.4 (36.2; 66.8)	52 (37; 68.8)	46.4 (32.1; 64.6)	47.1 (38.2; 66.4)	0.286
AST, U/L, Me (IQR)	45 (33; 62.5)	35.5 (28.7; 49.5)	45 (34; 66.7)	55 (41; 79)	<0.001
ALT, U/L, Me (IQR)	33 (25; 47.5)	37 (29; 51.2)	33 (25.2; 50.2)	32 (23; 44)	0.012
LVEF, %, Me (IQR)	40 (28; 53)	45 (34.2; 60)	42 (30; 52)	32 (20; 45)	<0.001
TAPSE, mm, Me (IQR)	19 (17; 20)	19 (17; 20)	19 (17; 20)	18 (16; 20)	0.305
IVC, mm, Me (IQR)	20 (19; 21)	19 (18.7; 21)	20 (19; 21.7)	21 (19; 22)	0.018
IVC ≥ 21 mm	100 (40.2)	22 (26.8)	36 (40.9)	42 (53.2)	0.003
Discharge medications, n (%)					
Beta-blocker	111 (44.6)	45 (54.9)	34 (38.6)	32 (40.5)	0.07
RAAS inhibitor	194 (77.9)	65 (79.3)	66 (75)	63 (79.7)	0.713
SGLT2i	121 (48.6)	41 (50)	41 (46.6)	39 (49.4)	0.894
MRAs	131 (52.6)	48 (58.5)	40 (45.5)	43 (54.4)	0.216
Loop diuretics	232 (93.2)	77 (93.9)	78 (88.6)	77 (97.5)	0.074

1 ADHF, acute decompensated heart failure; ALT, alanine aminotransferase; AST, aspartate aminotransferase; CAD, coronary artery disease; CKD, chronic kidney disease; CVA, cerebrovascular accident; eGFR, estimated glomerular filtration rate; IQR, interquartile range; IVC, inferior vena cava; LVEF, left ventricular ejection fraction; Me, median; MI, myocardial infarction; MRAs, mineralocorticoid receptor antagonists; NT-proBNP, N-terminal pro-B-type natriuretic peptide; NYHA, New York Heart Association; RAAS, Renin-Angiotensin-Aldosterone System; SGLT2i, SodiumGlucose Cotransporter-2 inhibitors; TAPSE, tricuspid annular plane systolic excursion.

With respect to discharge therapy, prescription rates of guideline-directed medical treatments were generally high and did not differ significantly among tertiles. Loop diuretics were prescribed in over 90% of patients across all groups. Use of renin-angiotensin-aldosterone system inhibitors and beta-blockers was common (approximately 75%-80% and 40%-55%, respectively), while mineralocorticoid receptor antagonists and sodium-glucose cotransporter-2 inhibitors were prescribed in about half of patients, without significant variation between tertiles.

**Figure 1.**
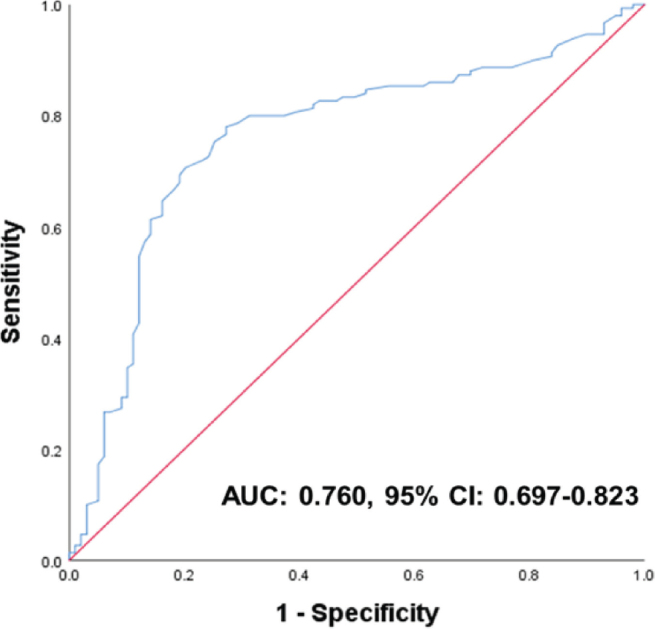
ROC curve of the De Ritis ratio for predicting 1-year MACEs in elderly patients hospitalized with acute decompensated HF. AUC, area under the curve; CI, confidence interval; HF, heart failure; MACEs, major adverse cardiovascular events; ROC, receiver operating characteristic.

At 1-year follow-up, 68 patients (27.3%) died, including 15 (6.0%) during hospitalization. MACEs occurred in 150 patients (60.2%). The incidence of MACEs increased progressively across De Ritis tertiles: 31.7% in the first tertile, 64.8% in the second, and 84.8% in the third (*P* < 0.001) (**Table 2**). ROC curve analysis identified a De Ritis ratio cutoff value of 1.30 for predicting 1-year MACEs, with an AUC of 0.760 (95% CI: 0.697-0.823, *P* < 0.001), a sensitivity of 78.0%, and a specificity of 72.7%. The corresponding positive and negative predictive values were 81.3% and 68.6%, respectively (**Figure 1**).

**Table 2.** One-year MACEs in the study population stratified by tertiles of the De Ritis ratio

Variables	Total cohort (n = 249)	De Ritis ratio			*P*
		1st tertile (n = 82)	2nd tertile (n = 88)	3rd tertile (n = 79)	
**All-cause death**					
In-hospital mortality, n (%)	15 (6)	2 (2.4)	9 (10.2)	4 (5.1)	0.094
1-year mortality, n (%)	53 (21.3)	7 (8.5)	19 (21.6)	27 (34.2)	<0.001
**MACEs**	150 (60.2)	26 (31.7)	57 (64.8)	67 (84.8)	
Overall 1-year mortality, n (%)	68 (27.3)	9 (11)	28 (31.8)	31 (39.2)	<0.001
HF rehospitalization, n (%)	79 (31.7)	15 (18.3)	27 (30.7)	37 (46.8)	
MI, n (%)	10 (4.0)	1 (1.2)	3 (3.4)	6 (7.6)	
Stroke, n (%)	15 (6.0)	0 (0)	11 (12.5)	4 (5.1)	

1 HF, heart failure; MACEs, major adverse cardiovascular events; MI, myocardial infarction.

**Figure 2.**
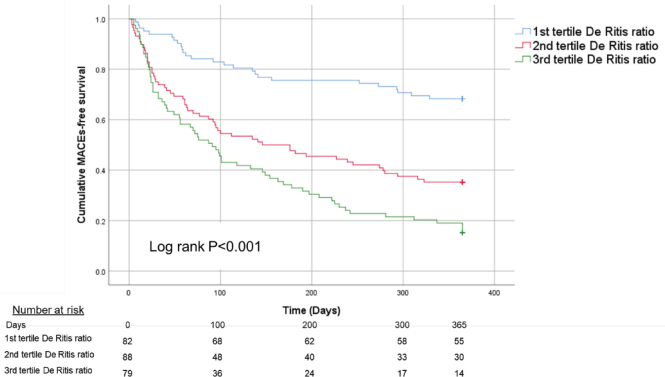
Kaplan-Meier survival curves for 1-year MACEs stratified by tertiles of the De Ritis ratio. MACEs, major adverse cardiovascular events.

Kaplan-Meier survival analysis demonstrated significantly lower MACEs-free survival in patients in the highest De Ritis tertile compared to the lower tertiles (log-rank *P* < 0.001). The unadjusted HR for MACEs per tertile increment of the De Ritis ratio was 2.02 (95% CI: 1.64-2.48, *P* < 0.001) (**Figure 2**). In multivariate Cox proportional hazards regression analysis, independent predictors of 1-year MACEs included diabetes mellitus (HR: 1.480; 95% CI: 1.011-2.167; *P* = 0.044), chronic lung disease (HR: 1.695; 95% CI: 1.010-2.845; *P* = 0.046), prior cerebrovascular accident (CVA) (HR: 1.497; 95% CI: 1.021-2.197; *P* = 0.039), log-transformed NT-proBNP (HR: 3.760; 95% CI: 2.066-6.844; *P* < 0.001), LVEF ≤ 40% (HR: 1.559; 95% CI: 1.057-2.298; *P* = 0.025), and a De Ritis ratio >1.30 (HR: 2.362; 95% CI: 1.536-3.634; *P* < 0.001) (**Table 3**).

**Table 3.** Cox proportional hazards regression analysis for predictors of 1-year MACEs

Variables	Univariate		Multivariate	
	HR (95% CI)	*P*	HR (95% CI)	*P*
Age (years)	1.026 (1.002-1.044)	0.003	1.002 (0.983-1.022)	0.820
Female	1.268 (0.918-1.751)	0.149	1.231 (0.859-1.765)	0.258
Arterial hypertension	1.185 (0.724-1.939)	0.500	0.967 (0.571-1.638)	0.901
CAD	1.930 (1.354-2.751)	<0.001	1.221 (0.820-1.819)	0.326
Atrial fibrillation	0.868 (0.629-1.197)	0.387	0.850 (0.605-1.194)	0.349
Diabetes mellitus	2.941 (2.126-4.068)	<0.001	1.480 (1.011-2.167)	0.044
Chronic lung disease	1.670 (1.052-2.652)	0.03	1.695 (1.010-2.845)	0.046
Previous CVA	2.348 (1.659-3.323)	<0.001	1.497 (1.021-2.197)	0.039
Anemia	1.286 (0.931-1.775)	0.127	1.154 (0.820-1.624)	0.412
logNT-proBNP	11.134 (7.098-17.456)	<0.001	3.760 (2.066-6.844)	<0.001
LVEF ≤ 40%	2.409 (1.726-3.363)	<0.001	1.559 (1.057-2.298)	0.025
eGFR < 60 mL/min/1.73 m^2^	1.572 (1.094-2.259)	0.014	1.010 (0.664-1.538)	0.961
De Ritis ratio >1.30	4.378 (2.962-6.472)	<0.001	2.362 (1.536-3.634)	<0.001

1 CAD: coronary artery disease; CI: confidence interval; CVA: cerebrovascular accident; eGFR: estimated glomerular filtration rate; HR: hazard ratio; LVEF: left ventricular ejection fraction; MACEs, major adverse cardiovascular events.

## Discussion

Our study investigated the prognostic value of the De Ritis ratio in elderly patients hospitalized with ADHF. The main finding was that an elevated De Ritis ratio at admission was independently associated with an increased risk of MACEs at 1 year. A cutoff value of 1.30 was identified as optimal for predicting adverse outcomes, with acceptable sensitivity and specificity. These results suggest that the De Ritis ratio may serve as a simple, accessible prognostic indicator in this high-risk population.

The underlying mechanism for this association is likely related to hepatic congestion and impaired perfusion secondary to elevated systemic venous pressure in ADHF. Increased venous congestion leads to hepatocellular atrophy and perisinusoidal edema, which reduces oxygen diffusion to hepatocytes, particularly in the centrilobular zone, the region most distant from the hepatic artery and portal vein, making it more susceptible to ischemic injury [18, 19]. Since AST is predominantly localized in this centrilobular region and is more sensitive to hypoxic damage than ALT, hepatic congestion tends to result in a greater increase in AST levels, thereby raising the AST/ALT (De Ritis) ratio [18, 19]. This mechanism is supported by our finding that higher De Ritis ratio was associated with markers of systemic congestion such as increased IVC diameter, elevated NT-proBNP levels, and lower LVEF. In addition, AST is also present in other tissues, including cardiac and skeletal muscles [20]. Therefore, elevated AST levels may also reflect subclinical myocardial injury or systemic catabolic processes, both of which are known to contribute to worse prognosis in patients with HF [21, 22]. This observation may partly explain the strong and independent prognostic value of the De Ritis ratio, even after adjustment for NT-proBNP and other clinical variables.

### Component outcomes and liver-injury signal

In our elderly ADHF cohort, higher admission De Ritis ratio was associated with a graded increase in several 1-year components: all-cause death (8.5%, 21.6%, 34.2%), HF rehospitalization (18.3%, 30.7%, 46.8%), and MI (1.2%, 3.4%, 7.6%) across the first to third tertiles, respectively. Stroke exhibited a nonmonotonic pattern (0%, 12.5%, 5.1%). These findings support a link between greater cardio-hepatic stress and both mortality and morbidity, particularly recurrent decompensation (rehospitalization). Notably, inhospital mortality was numerically lower in the third versus second tertile (5.1% vs 10.2%), but this difference was not statistically significant (*P* = 0.094) and involved small event counts; it is therefore most likely explained by random variation and case-mix effects rather than a true protective association. Overall, the clearest and most consistent gradient in our study was seen for HF rehospitalization and total MACE burden, suggesting that the De Ritis ratio reflects overall disease severity through a combination of hepatic congestion, systemic hypoperfusion, and extrahepatic AST release, rather than being linked to a single outcome.

The prognostic role of the De Ritis ratio in HF has been evaluated in several prior studies. Maeda et al. [10], in a cohort of 774 elderly patients (mean age 80.2 ± 7.8 years, 43% female) hospitalized for ADHF, demonstrated that a De Ritis ratio ≥1.7 was an independent predictor of 1-year mortality, with an AUC of 0.60 (95% CI: 0.56-0.65). In comparison, the AUC in our study was 0.76 (95% CI: 0.70-0.82) in predicting MACEs, indicating better discriminative ability in predicting adverse outcomes in elderly Vietnamese patients with ADHF. In a cohort of 3,212 patients with HF with preserved ejection fraction, Cao et al. reported that a De Ritis ratio >1.0 was associated with a 30% increased risk of MACEs over a median follow-up of 3.3 years [12], reinforcing the prognostic value of this biomarker in different HF phenotypes. Additionally, the ESC-HF-LT Registry, a large prospective, multicenter cohort of 9,134 HF patients, identified liver dysfunction as an independent predictor of 1-year all-cause mortality in patients with mildly reduced LVEF, with an adjusted odds ratio of 2.37 (95% CI: 1.28-4.38, *P* = 0.0059) [23]. This finding highlights the clinical importance of incorporating hepatic parameters, including the De Ritis ratio, into risk assessment models for HF patients.

A recent comprehensive review by Ndrepepa [24] summarized accumulating evidence regarding the prognostic implications of the De Ritis ratio in cardiovascular diseases. Elevated De Ritis ratio has been independently associated with increased risk of cardiovascular mortality in multiple cohorts, including patients with hypertension [25], CAD [26], and acute MI [27]. In CAD populations, Ndrepepa et al. [26] reported that in 5,020 patients with stable CAD undergoing percutaneous coronary intervention, all-cause mortality rates at 3 years were 5.0%, 7.5%, and 14.5% across De Ritis ratio tertiles, with an adjusted HR of 1.09 (95% CI: 1.06-1.12, *P* < 0.001) per unit increase, and similar trends for cardiovascular death. In a pooled analysis of high-risk diabetic patients, Ferrannini et al. [28] reported that each unit increase in the log De Ritis ratio was associated with a 2-fold increase in the risk of HF hospitalization and cardiovascular death. These findings confirm the prognostic value of the De Ritis ratio in various cardiovascular settings.

The prognostic value of the De Ritis ratio remained significant after adjusting for NT-proBNP and LVEF, 2 of the most widely accepted prognostic markers in HF. This is of particular interest, given that access to NT-proBNP testing remains limited in many low- and middle-income countries. The De Ritis ratio, derived from routine liver function panels, offers a low-cost, widely available alternative or adjunct to established biomarkers, particularly in resource-constrained settings such as Vietnam.

In the multivariable Cox regression analysis, several clinical and laboratory variables were identified as independent predictors of 1-year MACEs in our study. Type 2 diabetes mellitus (HR: 1.48), chronic lung disease (HR: 1.70), and previous CVA (HR: 1.50) were independently associated with adverse outcomes, consistent with previous studies [29, 30]. Elevated log-transformed NT-proBNP (HR: 3.76) and LVEF ≤ 40% (HR: 1.56) remained important prognostic indicators, reaffirming their established value in acute HF [31]. Notably, an elevated De Ritis ratio >1.30 retained its independent predictive value (HR: 2.36, *P* < 0.001) after adjustment for these variables, underscoring its potential as a complementary biomarker reflecting systemic congestion, multiorgan dysfunction, and disease severity.

This study has several limitations. First, it was conducted at a single specialized cardiovascular center, which may limit the generalizability of its findings to other patient populations or healthcare settings. Second, we analyzed only the baseline De Ritis ratio at hospital admission; serial measurements may have provided a more accurate assessment of dynamic hepatic or systemic congestion during hospitalization and follow-up. Third, although patients with known chronic liver disease were excluded, the influence of unrecognized hepatic dysfunction or alcohol use cannot be fully ruled out. Fourth, despite adjustment for major clinical covariates, residual confounding from unmeasured factors—such as nutritional status, sarcopenia, or frailty—may have affected the results. Fifth, the study was not powered to perform cause-specific analyses of individual MACE components; thus, subgroup findings, particularly for inhospital mortality and stroke, should be interpreted cautiously. Finally, although we identified an optimal cutoff value of 1.30 for predicting adverse events, this threshold requires external validation in larger, multicenter cohorts before it can be adopted in routine clinical practice.

## Conclusion

An elevated De Ritis ratio was independently associated with an increased risk of 1-year MACEs in elderly patients hospitalized with ADHF. This simple and accessible biomarker may provide additional prognostic value for clinical risk stratification in this high-risk population.
